# Cumulative Negative Impacts of Invasive Alien Species on Marine Ecosystems of the Aegean Sea

**DOI:** 10.3390/biology12070933

**Published:** 2023-06-29

**Authors:** Konstantinos Tsirintanis, Maria Sini, Michail Ragkousis, Argyro Zenetos, Stelios Katsanevakis

**Affiliations:** 1 Department of Marine Sciences, University of the Aegean, Lofos Panepistimiou, 81100 Mytilene, Greece; 2 Hellenic Centre for Marine Research (HCMR), Institute of Marine Biological Resources and Inland Waters, 19013 Attika, Greece

**Keywords:** non-indigenous species, exotic species, cryptogenic species, impacts, CIMPAL, biodiversity, mapping, management, Mediterranean Sea

## Abstract

**Simple Summary:**

Human activities and interventions, such as creating sea basin connections (e.g., the Suez Canal), shipping, and aquaculture can serve as pathways for marine species introductions to new ecosystems beyond their natural geographical range. These species are called aliens and may become invasive, negatively impacting the recipient ecosystem. The effective management of invasive species requires understanding and assessing their impacts on the native biota. In this study, we mapped and quantified the cumulative impacts of invasive species on the marine habitats of the Aegean Sea, a Mediterranean ecoregion that is heavily affected by biological invasions. Our findings show that coastal habitats were more impacted by invasive species than the open sea. A higher frequency of strong impacts was observed in the South Aegean compared to the North Aegean, primarily due to alien fish and macrophytes. Shallow hard substrates were the most impacted habitat type, followed by shallow soft substrates and seagrass meadows. The worst invasive species varied depending on the habitat type and impact indicator used. Our study aligns with European guidelines for managing invasive species’ impacts on native biodiversity and can serve as an essential tool for managing biological invasions and mitigating their impacts in the Aegean Sea.

**Abstract:**

Biological invasions are a human-induced environmental disturbance that can cause major changes in ecosystem structure and functioning. Located in the northeastern Mediterranean basin, the Aegean Sea is a hotspot of biological invasions. Although the presence of alien species in the Aegean has been studied and monitored, no assessment has been conducted on their cumulative impacts on native biodiversity. To address this gap, we applied the CIMPAL index, a framework developed for mapping the cumulative impacts of invasive species, to identify the most affected areas and habitat types and determine the most invasive species in the region. Coastal areas showed stronger impacts than the open sea. The highest CIMPAL scores were four times more frequent in the South than in the North Aegean. Shallow (0–60 m) hard substrates were the most heavily impacted habitat type, followed by shallow soft substrates and seagrass meadows. We identified *Caulerpa cylindracea*, *Lophocladia lallemandii*, *Siganus luridus*, *Siganus rivulatus,* and *Womersleyella setacea* as the most impactful species across their range of occurrence in the Aegean but rankings varied depending on the habitat type and impact indicator applied. Our assessment can support marine managers in prioritizing decisions and actions to control biological invasions and mitigate their impacts.

## 1. Introduction

Human activities are causing significant changes in the oceans, leading to unprecedented disruptions in natural processes [1,2,3]. Biological invasions are regarded as one of the most severe human-induced disturbances as they reshape the structure and functioning of ecosystems worldwide [4,5]. The rate of alien species’ introductions has accelerated since the 19th century [6] and is projected to intensify further in the 21st century, particularly in Europe [7]. Even under optimistic scenarios, the impacts of alien species on biodiversity will continue to grow, with significant consequences for communities and ecosystems [8].

Biological invasions are an integral part of the Anthropocene and contribute to global change [9]. There are ways to prevent new biological invasions by managing introduction pathways and mitigating the impacts of established species through targeted actions focusing on harmful species and priority areas [5,8]. However, managing marine alien species poses several challenges, including accurately assessing their negative impacts and their magnitude [10,11]. Not all alien species are invasive (i.e., have negative impacts on biodiversity, ecosystem services, or human health). Several established alien species have positive impacts, such as providing food for native species or humans, creating new habitats, and controlling other alien species or population explosions [10,12]. The impacts of alien species can vary across space [13] or time [14] and are greatly affected by the level of co-occurrence and interaction with other human disturbances [15,16,17]. Uncertainties in predicting the distribution of alien species and determining the factors that define habitat invasibility, vulnerability, and resilience undermine the reliability of bioinvasion studies and hinder decision-making for specific management actions [18]. Additionally, research on bioinvasions is limited by weak evidence, frail correlations, and a lack of experimental studies [12,19].

Despite these limitations, the utilization of impact mapping techniques offers a promising tool to tackle the crisis of biodiversity loss and prioritize relevant management actions [20,21,22]. Although demanding, these methodologies effectively identify areas receiving the highest impact pressure, enabling marine managers to establish priorities [23,24]. Impact mapping has been successfully applied at various spatial scales, including the Mediterranean Sea, and has even been utilized to map the impacts of invasive species [13,25].

The Mediterranean Sea has the highest number of alien species globally [26], which has caused significant adverse impacts on native marine biodiversity [12,13]. Over 1000 marine alien species have been introduced into the Mediterranean, with more than 750 having established populations [27,28]. Additionally, the rate of establishment of marine alien species in the Mediterranean is accelerating, with no signs of leveling off [27,29]. The actual number of established alien species in the Mediterranean is probably underestimated due to the exclusion of cryptogenic species. These are species of unknown biogeographic origin that could be either alien or native [30,31]. The number of cryptogenic species in the Mediterranean Sea is estimated to be 58 taxa [27]. Neglecting established cryptogenic species when assessing the impacts of biological invasions can result in underestimated invasion impacts on biological communities [30]. Tsirintanis et al. [12] evaluated the impacts of alien and cryptogenic species in the Mediterranean, identifying 71 alien and 5 cryptogenic species with significant negative effects on native biodiversity, ecosystem services, and human health.

The eastern Mediterranean Sea is particularly vulnerable to biological invasions, with most new introductions occurring there [29,32]. The region’s overall warmer climatic conditions, faster pace of sea-water temperature rise, and the opening of the Suez Canal enhance the introduction, establishment, and impacts of thermophilous Lessepsian immigrants on native ecosystems [33,34,35]. Located in the northeastern part of the Mediterranean Sea, the Aegean Sea is an ecoregion [36] that supports rich biodiversity but lacks effective conservation management of its marine resources [37]. By 2019, 209 alien species had been recorded in the Aegean, with 149 having established populations [38]. Among the latter, 48 are considered to be invasive [12]. Accumulating evidence over the last 20 years in the area indicates an alarming increase in the abundance of records and the number of reported impactful cryptogenic and alien species (ICAS) [39].

Local climatic conditions, along with other human activities that enhance the introduction of alien species, their natural expansion and their impacts, render the Aegean an ecoregion under siege that urgently needs effective management measures to eliminate local biodiversity loss within a proactive and adaptive systematic conservation planning approach. Such an approach requires continuous monitoring of alien species distribution, introduction pathways, and assessment of their impacts [38,40]. Although the presence of alien species in the Aegean Sea has been monitored over the past two decades [41,42], through scientific networks [43,44], citizen science initiatives [45], and collections of unpublished records [46,47], and their introduction pathways have been assessed [33,48], there are no studies that address their cumulative impacts under a systematic framework.

To address this gap and assess the impacts of marine ICAS in the Aegean Sea, we used the CIMPAL index (cumulative impacts of invasive alien species), a framework developed for mapping the cumulative negative impacts of marine alien species on marine habitats [13]. Our goal was to identify highly impacted areas and habitat types, rank the ICAS by their impacts, and assist the prioritization of future management decisions and actions to control and mitigate marine bioinvasions in the region.

## 2. Materials and Methods

We employed the CIMPAL index to assess and map the effects of biological invasions in the Aegean Sea, specifically focusing on the impacts of 26 ICAS (Table 1). The selection of the specific ICAS was based on two criteria; (a) having negative impacts on Mediterranean biodiversity [12], and (b) having a substantial number of presence records (Table 1) in the Aegean Sea based on the datasets provided by Ragkousis et al. [39,47]. For the purpose of the analyses, targeted ICAS were classified into three groups: invertebrates, macrophytes, and fish. The foraminifer *Amphistegina lobifera* was included in invertebrates (Table 1).

To assess the ICAS’ impacts, we partitioned the Aegean Sea into 0.01° latitude/longitude grid cells and estimated the percent cover of ten broad habitat types in each cell, based on habitat maps produced by Sini et al. [37] and Topouzelis et al. [49]. The ten habitat types (Figures S1–S10) were: (1) seagrass meadows, (2) shallow soft substrates (0–60 m depth), (3) deep soft substrates (60–200 m depth), (4) soft substrates of the dysphotic zone (deeper than 200 m), (5) shallow hard substrates (0–60 m depth), (6) deep hard substrates (60–200 m), (7) hard substrates of the dysphotic zone (deeper than 200 m), (8) submarine caves, (9) coralligenous formations, and (10) the pelagic habitat. For the analyses, we classified habitat types at depths of 0–200 m as coastal and those deeper than 200 m as open sea habitats. The pelagic habitat was considered part of both coastal and open sea habitats and covered the entire study area.

The CIMPAL index was calculated for each grid cell of the study area using the formula $I_{c}=\overset{n}{\underset{i=1}{\sum }}\overset{m}{\underset{j=1}{\sum }}A_{i}H_{j}w_{i,j}$. In this formula, *A_i_* refers to the population state of species *i* in a given cell of the study area, standardized to range between 0 and 1. We estimated *A_i_* for each targeted ICAS (Table 1), using two approaches: (a) species distribution models (SDMs) within the Aegean, derived by Ragkousis et al. [50], and (b) ICAS presence/absence data acquired from Ragkousis et al. [39,47]. *H_j_* is the percent cover of habitat *j* in a given cell of the study area. The impact weight *w_i,j_* of ICAS *i* at habitat type *j* was estimated using an uncertainty-averse approach [13,51] (Figure S11) based on both the species impact magnitude (Figure S12; according to Blackburn et al. [11] and Volery et al. [52]) and the strength of the reported evidence (Figure S12 according to Katsanevakis et al. [19]). The impact magnitude and strength of evidence for every combination of ICAS and habitat type (Table S1) were retrieved from Tsirintanis et al. [12].

SDMs [50] were produced using the “biomod2” R package and an ensemble modeling approach [53,54]. Presence data were collected through both field work and an extensive literature review in the framework of the ALAS project [39,40]. The environmental predictors used included temperature (min, max, mean) and chlorophyll-a for the period 2019–2021, which were retrieved from the COPERNICUS Marine Environment Monitoring Service. Potential collinearity was assessed using the variance inflation factor (VIF) [55]. A set of random pseudo-absences were generated for each species, containing the same number of data points as the species’ presences, located at distances greater than 100 km from known presences. Five algorithms were implemented: generalized linear models (GLMs), generalized additive models (GAMs), classification tree analysis (CTA), random forest (RF), and multivariate adaptive regression spline (MARS). Model performance was evaluated using a three-times 70–30% data splitting method, and evaluated via their true skill statistics (TSS) score [56]. Only models with a TSS value > 0.6 were included in the ensemble models [57].

To rank the ICAS based on their negative impacts, we estimated four different indicators: (D1) the total area of an ICAS occurrence, which was calculated as the total number of grid cells with at least one record of the specific ICAS; (D2) the number of cells with an estimated impact score > 0 for a given ICAS; (D3a) the sum of impact score values of a specific ICAS across the entire study area based on SDMs; (D3b) the sum of impact score values of a specific ICAS across the entire study area based on species records; (D4) the average impact score across the ICAS’s range of occurrence, excluding cells with score values lower than 0.1.

## 3. Results

**Figure 1:**
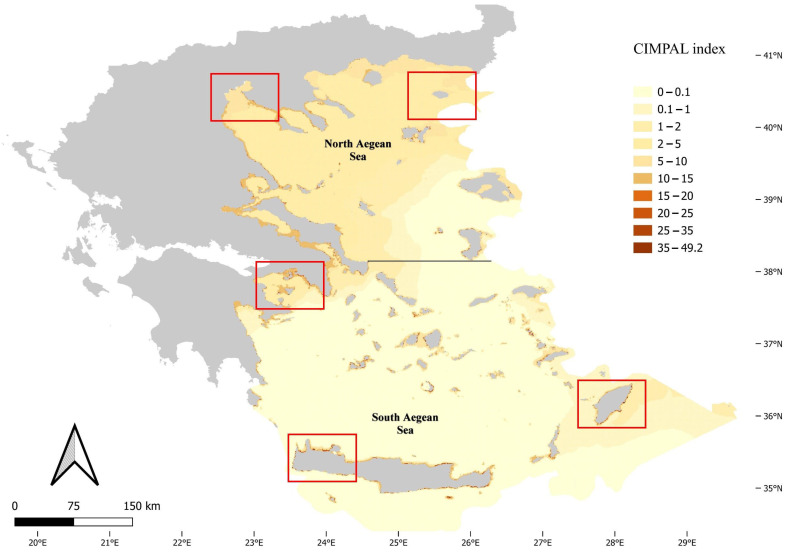
Map of ICAS cumulative impacts in the Aegean Sea, according to the CIMPAL index scores. Red rectangles highlight selected areas depicted in Figure 2.

**Figure 2:**
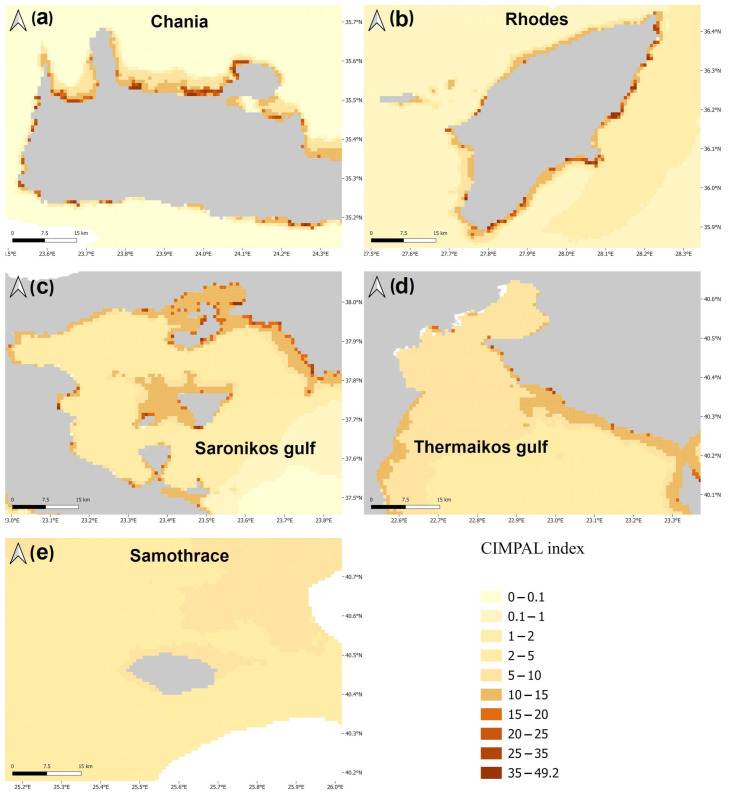
Examples depicting the variation in CIMPAL scores in the Aegean Sea. Notably the South Aegean areas (**a**–**c**) display a higher frequency of high CIMPAL scores compared to the North Aegean areas (**d**,**e**).

The use of the CIMPAL index unveiled the spatial patterns of ICAS negative cumulative impacts in the Aegean Sea (Figure 1). The study area was divided into 321,346 grid cells, with the majority of cells (69%) corresponding to the open sea, and only 31% to coastal habitat types. Cumulative CIMPAL impact scores per cell ranged from 0 to 49.2, with a mean of 1.35. Coastal habitat types found at depths up to 60 m exhibited higher CIMPAL impact scores compared to deeper coastal habitats or the open sea (Figure 1). In the open sea, cumulative impacts decreased from North to South Aegean (Figure 1). However, the highest CIMPAL scores (>20) only occurred in shallow coastal ecosystems (up to 60 m depth) and were localized without covering extensive surface areas. These high scores were four times more frequent (79%) in the South compared to the North Aegean (Figure 1 and Figure 2).

**Figure 3:**
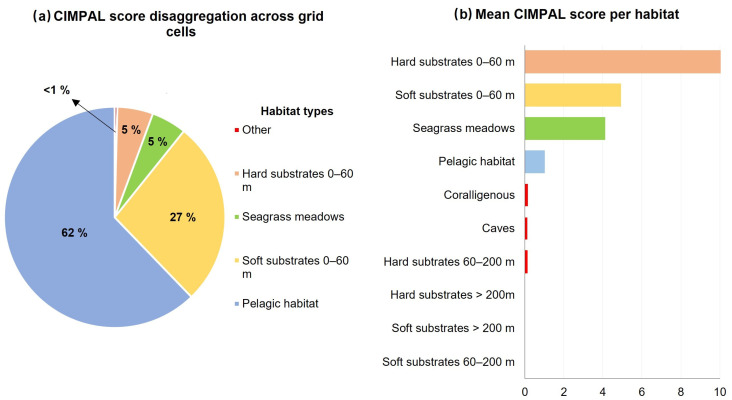
(**a**) Disaggregation of the total CIMPAL score per habitat type, (**b**) standardized mean CIMPAL scores per habitat type.

The pelagic habitat had the greatest contribution to the overall sum of CIMPAL score for all grid cells in the Aegean Sea; although, it was only impacted by a single ICAS, the alien ctenophore *Mnemiopsis leidyi* (Figure 3a). This high contribution was due to the extensive coverage of this habitat type (Figure S10) rather than because of the per-cell values of the CIMPAL index, which ranged between 0 and 4.0, with a mean of 1.0 (Figure 3b).

**Figure 4:**
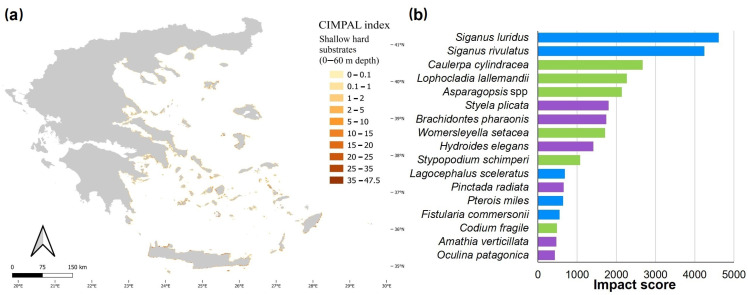
(**a**) Estimation of the CIMPAL index for shallow hard substrates (0–60 m) in the Aegean Sea. (**b**) ICAS in this habitat type, ranked by the estimated total impact score per species (summed over all grid cells). Green color for macrophytes, blue for fish and purple for invertebrates.

Shallow hard substrates (0–60 m depth) covered only 0.1% of the study area and accounted for 5% of the total CIMPAL score (Figure 3a). This habitat type displayed the widest range of CIMPAL scores (0 to 47.5) and was identified as the most impacted habitat type, with a mean CIMPAL score value of 10.6 (Figure 3b). High scores appeared more frequently in the South than North Aegean (Figure 4a). In total, 17 invasive alien species negatively affected this habitat, with *Siganus luridus*, *Siganus rivulatus*, and *Caulerpa cylindracea* being the most impactful (Figure 4b).

**Figure 5:**
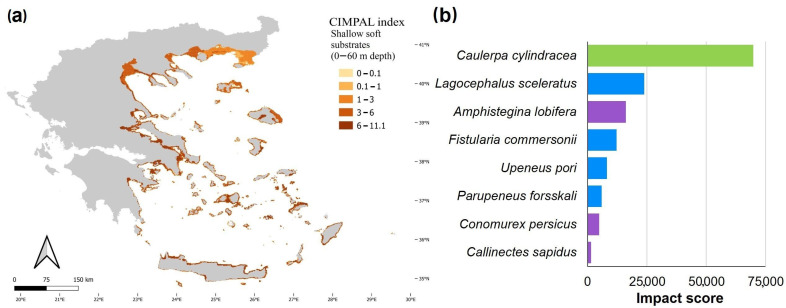
(**a**) Estimation of the CIMPAL index for shallow soft substrates between 0 and 60 m depth in the Aegean Sea. (**b**) ICAS in this habitat type, ranked by the estimated total impact score per species (summed over all grid cells). Green color for macrophytes, blue for fish and purple for invertebrates.

Shallow soft substrates (0–60 m depth) covered 6.5% of the Aegean Sea’s surface and accounted for 27% of the total CIMPAL scores (Figure 3a). The CIMPAL impact scores for this habitat type ranged from 0 to 11.1, with a mean value of 4.9, ranking shallow soft substrates as the second most impacted habitat type (Figure 3b). Scores were higher in the South than the North Aegean (Figure 5a). This habitat type was impacted by eight invasive species, with *C. cylindracea* having the highest impact, followed by the invasive fish *Lagocephalus sceleratus*, and the foraminifer *A. lobifera* (Figure 5b). 

**Figure 6:**
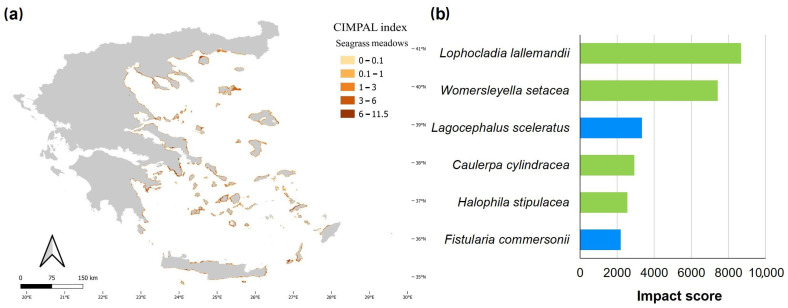
(**a**) Estimation of the CIMPAL index for seagrass meadows in the Aegean Sea. (**b**) ICAS in this habitat type, ranked by the estimated total impact score per species (summed over all grid cells). Green color for macrophytes, blue for fish and purple for invertebrates.

Seagrass meadows covered 0.8% of the study area’s surface and accounted for 5% of CIMPAL scores (Figure 3a). Impact values ranged from 0 to 11.5 and had a mean of 4.2 (Figure 3b). The CIMPAL score exhibited increased heterogeneity across the study area, without clear geographical patterns (Figure 6a). The red algae *Lophocladia lallemandii* and *Womersleyella setacea* were the invasive species with the highest negative impact on seagrass meadows (Figure 6b). 

Impacts on marine caves, coralligenous formations, and hard substrates between 60 and 200 m depth accounted for less than 1% of the total of CIMPAL scores in the Aegean (Figure 3a). No species impacting the rest of the habitat types were documented.

**Figure 7:**
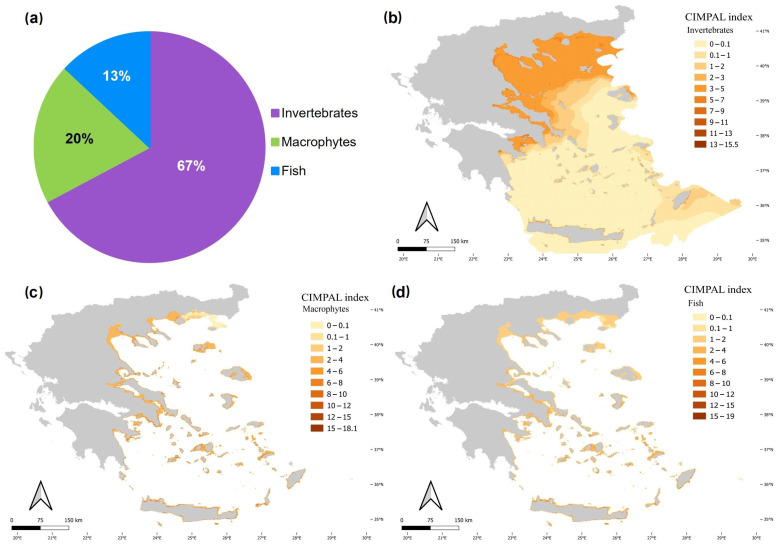
(**a**) Disaggregation of the total CIMPAL score by biotic group (invertebrates: purple color, macrophytes: green, fish: blue), and maps of ICAS impacts in the Aegean Sea for (**b**) invertebrates, (**c**) macrophytes, and (**d**) fish.

Invertebrates were the biotic group with the highest contribution to the cumulative ICAS impacts in the Aegean Sea, accounting for 67% of the total CIMPAL score (Figure 7a). In the open sea, their impacts showed a decreasing trend from the North to the South Aegean (Figure 7b). This is due to the invasive ctenophore *M. leidyi* (92% of the invertebrate CIMPAL score), which is the only species affecting the pelagic habitat (i.e., the habitat type with the greatest cover). Macrophytes accounted for 20% of the total CIMPAL score (Figure 7a). Their impact did not show any evident spatial pattern across the study area, apart from the lower scores in the northeastern Aegean (Figure 7c). Macrophytes’ higher CIMPAL scores (>10) were more frequent (76%) in the South than in the North Aegean Sea. Fish accounted for 13% of the cumulative impacts in the region (Figure 7a) and had stronger impacts on the coastal habitats of the South Aegean (Figure 7d); high CIMPAL scores attributed to fish (>10) were much more frequent in the South Aegean (88%).

**Figure 8:**
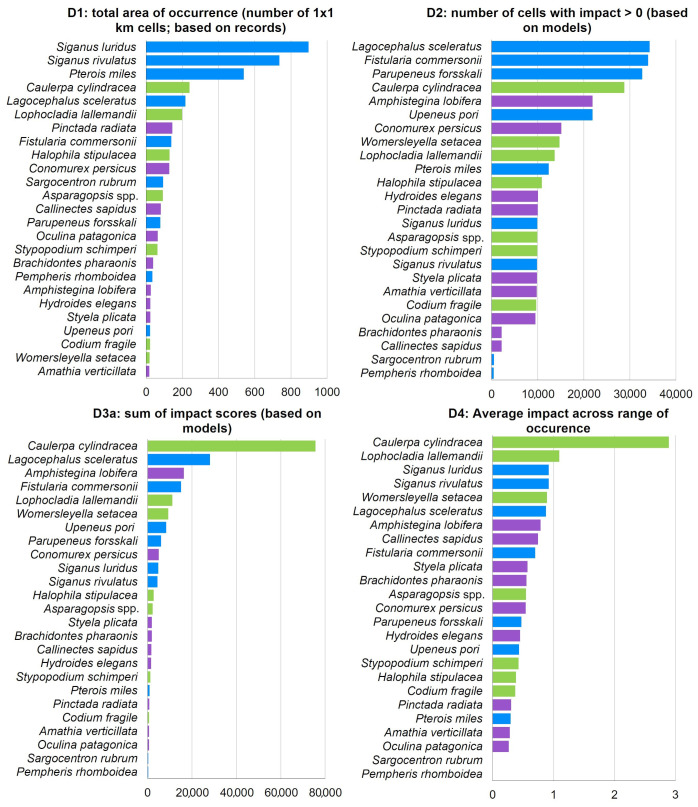
Ranking of ICAS (excluding *Mnemiopsis leidyi*) impacts in the Aegean Sea according to the four applied indicators D1–D4. Macrophytes: green color, fish: blue, and invertebrates: purple.

The ICAS impact ranking varied depending on the indicator used (Figure 8). Excluding *M. leidyi* from all rankings, the herbivorous fishes *S. luridus* and *S. rivulatus*, along with the lionfish *Pterois miles*, were the top three ranking species according to D1 (total area of occurrence). According to D2 (number of cells with impact), the fishes *L. sceleratus*, *Fistularia commersonii*, and *Parupeneus forsskali* were the top three ranking species. According to D3a (sum of impact scores based on models), the green alga *C. cylindracea* was identified as the most impactful ICAS in the Aegean, with an impact score approximately three times higher than the second-ranking species, *L. sceleratus*. The top 10 most impactful invasive species, according to D3a, included five fish, three macrophytes, and two invertebrates (Figure 8). *Caulerpa cylindracea* was also ranked as the most impactful invasive species in the Aegean by indicator D4 (average impact across the range of occurrence), with a significant difference from the second-ranking *L. lallemandii*. D2 and D3a exhibited high similarity, with nine common species in their top ten (Figure 8), although with different rankings.

**Figure 9:**
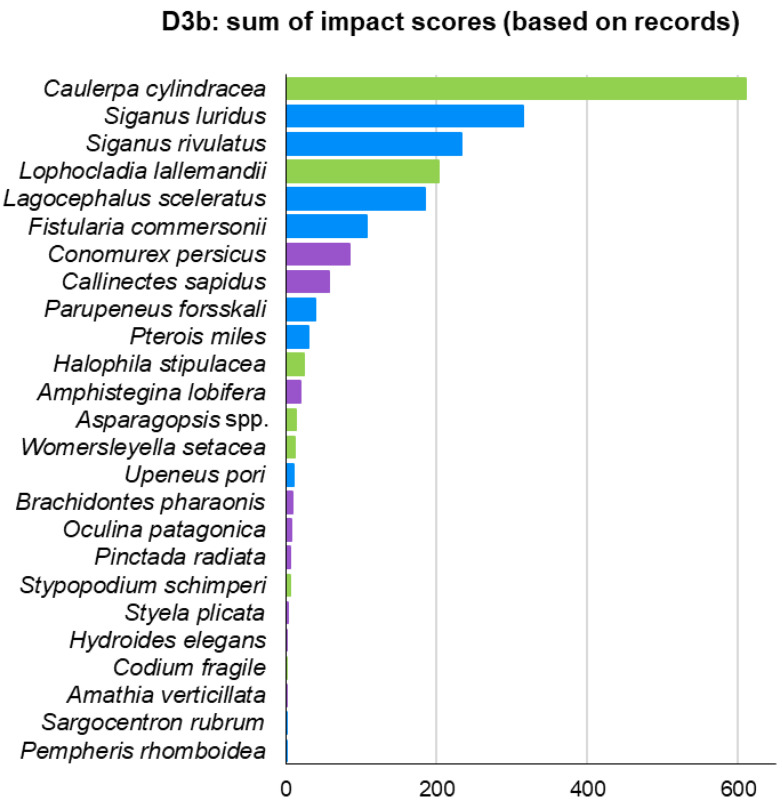
Ranking of ICAS (excluding *Mnemiopsis leidyi*) impacts in the Aegean Sea using indicator D3 with presence/absence data derived from field observations instead of modelled data.

Moreover, indicator D3 was also estimated based on actual ICAS records—D3b—(Figure 9) instead of their modelled distribution—D3a—(Figure 8). D3b ranked six fish, two macrophytes, and two invertebrates in its top 10. Although both methods had seven species in common in their top 10, there were some differences in the rankings. For instance, the two invasive siganids ranked 10th and 11th in D3a but ranked 2nd and 3rd in D3b. The invasive foraminiferan *A. lobifera* ranked 3rd in D3a and 12th in D3b. The lionfish *P. miles* ranked 19th in D3a and 10th in D3b (Figure 8 and Figure 9).

## 4. Discussion

According to the CIMPAL index, the impact scores of the ICAS in the Aegean Sea are higher in coastal habitat types compared to the open sea. The invasive ctenophore *Mnemiopsis leidyi* is the only ICAS that extends its distribution to the open sea, whereas the impacts of the other ICAS appear to be confined to coastal habitats. Even within coastal waters (0–200 m depth), CIMPAL scores were higher in habitat types shallower than 60 m depth. Apart from *M. leidyi*, the only other ICAS found to impact coastal habitats deeper than 60 m was *P. miles*. In a pan-Mediterranean biodiversity assessment, Coll et al. [58] indicated that shallow coastal waters have higher biodiversity values and a higher concentration of alien species. Additionally, in the application of the CIMPAL index at the Mediterranean scale, Katsanevakis et al. [13] also reported that ICAS mainly impacted coastal habitats. These results may be attributed to the fact that most alien species introduced in the Mediterranean Sea are demersal or benthic species that prefer shallower and warmer waters [33].

However, the observed patterns of some ICAS impacts may partly reflect the spatial distribution of research efforts, which are primarily conducted in shallow coastal ecosystems (e.g., rocky reefs and seagrass meadows found in waters shallower than 60 m depth) as these habitats are more easily accessible to scientific divers and citizen scientists. Furthermore, research often focuses on areas that represent important introduction pathways (e.g., large ports) or that are systematically monitored by scientific institutes or other marine conservation agencies [13,33,39]. This research effort bias is a commonly reported problem in studies assessing biodiversity in the Mediterranean Sea. Marine research in the region has traditionally been restricted to shallow waters, whereas large parts of the deep sea remain understudied [37,58]. In the present study, the estimated cumulative impacts in the open sea of the Aegean are shaped by the modelled spatial distribution of *Mnemiopsis leidyi* in the region. However, the majority of the relevant data were collected during early summer surveys from 2004–2006 and 2008 conducted only in the North Aegean Sea [59]. As a result, the SDM depicted its occurrence throughout the Aegean heavily based on the research effort of the latter study; although, its presence in the South Aegean remains largely unknown. Considering that the species has been reported as far south as Syria and Israel [60,61], its presence in the South Aegean is possible. Furthermore, there is significant uncertainty regarding the origin, distribution, and impacts of several other planktonic or hard-to-identify ICAS. As a result, these species cannot be included as alien species in official lists [18,62,63,64].

The CIMPAL index results revealed the cumulative impacts of ICAS on coastal habitats throughout the Aegean Sea, with a higher frequency of stronger impacts occurring more frequently in the South Aegean. Impact mapping of the distinct biotic ICAS groups (Figure 7), showed that invasive fish were the primary cause of the higher coastal impact scores in the South. Recent studies on the spatiotemporal distribution patterns of marine ICAS throughout the Greek Seas have reported similar patterns, showing a higher ICAS richness and abundance of records in the South Aegean areas and gradually decreasing towards the North [33,39]. These distribution trends are believed to be related to species-specific ecological traits and introduction pathways [33,65]. For instance, most alien fish in the Mediterranean are thermophilous species of Indo-Pacific origin, introduced through the Suez Canal in the southeastern part of the Mediterranean [33]. Consequently, they are favored by the higher mean sea water temperatures of the South Aegean [66] and thrive in these ecosystems. 

Macrophytes’ impacts did not show specific large-scale spatial patterns across the study area, except for a higher occurrence of high CIMPAL scores in the South Aegean. The overall homogenous distribution of macrophytes’ impacts in the Aegean Sea is possibly attributed to the contrasting and heterogeneous spatial distributions of species within this biotic group. For example, the invasive chlorophyte *Codium fragile* is primarily recorded in the North Aegean, whereas the alien seagrass *Halophila stipulacea* and the invasive ochrophyte *Stypopodium schimperi* are mainly found in the South Aegean. Other species, such as *C. cylindracea* and *L. lallemandii*, are abundant in both the North and South Aegean Sea [39,50]. Katsanevakis et al. [33] depicted a heterogeneous spatial distribution of alien macrophytes in the Aegean Sea. In that study, aquaculture and shipping were reported as the two main pathways for macrophyte introduction in the Mediterranean Sea, indicating no common thermal affinity contrary to the case of Lessepsian species. The varying distribution patterns of macrophytes and their associated impacts are more likely related to their different species-specific ecological traits and niches, such as their varying thermal preferences [67,68]. 

Invertebrate ICAS impacts were generally higher in coastal habitats compared to the open sea, particularly in the South Aegean. Katsanevakis et al. [33] identified the Suez Canal and shipping as the two main pathways for invertebrate introductions in the Mediterranean Sea. Therefore, the pool of alien invertebrate species in the region includes both thermophilic species from the Red Sea that tend to be more abundant in the South Aegean and vessel-introduced species with various thermal preferences. The latter are often observed in or near ports [39]. Shipping and ports have been globally associated with bioinvasions [69,70], and shipping is regarded as one of the primary introduction pathways in the Mediterranean Sea [71,72,73]. Invertebrates account for a significant portion of the alien diversity within Mediterranean ports [74]. The Saronikos Gulf, located in the South Aegean Sea, is considered a hotspot for biological invasions due to Piraeus, the largest Greek port [73]. Several of the herein assessed invertebrate ICAS, such as *Styela plicata*, *Brachidontes pharaonis*, *Hydroides elegans*, *Pinctada radiata*, and *Amathia verticillata*, are found in high abundance within Mediterranean ports [71], and some of the depicted CIMPAL impacts in the Aegean are related to them. 

Shallow hard and soft substrates between 0 and 60 m depth and seagrass meadows were assessed as the coastal habitats with the highest mean CIMPAL impact score. Shallow hard substrates between 0 and 60 m depth were the most negatively impacted habitat type. The main species responsible for the impacts on this habitat type were the invasive herbivorous fish *S. luridus* and *S. rivulatus*. Several studies in the Aegean have linked the presence of *Siganus* spp. with adverse grazing effects on macroalgal communities [15,75,76,77,78] and a degraded rocky reef ecological state [79,80]. Three macroalgal ICAS follow in the list of the most impactful species on shallow hard substrates: the green alga *C. cylindracea*, and the red algae *L. lallemandii* and *Asparagopsis* spp. *Caulerpa cylindracea* impacts biodiversity in these habitats through various mechanisms such as competition, overgrowth, increased sedimentation, and ecosystem engineering [12]. The two invasive red algae can adversely impact native species through competition for resources [81,82,83] and through the creation of novel habitats that alter benthic community composition to a more impoverished state [84,85]. The invasive ascidian *S. plicata* was identified as the most impactful invertebrate ICAS on this habitat type. It forms dense aggregations that can cover the available hard substrate, which outcompete native species [86]. 

Soft substrates between 0 and 60 m depth exhibited the second highest sum of cumulative impacts and the second highest mean impact score value among the studied habitat types. *Caulerpa cylindracea* was the only macrophyte among the eight ICAS impacting shallow soft substrates, and ranked first as the most impactful species for this habitat type. The invasive green alga can form dense mats on shallow sediments that lead to major structural changes in the resident soft-bottom faunal communities and ecosystem functioning [87,88,89]. Half of the ICAS impacting soft substrates between 0 and 60 m depth were fish. *Lagocephalus sceleratus* and *F. commersonnii* are considered among the most impactful alien fish in the Mediterranean, being voracious predators that prey upon several native species [90,91,92,93]. Ecological modelling approaches have also indicated that *L. sceleratus* is an important competitor of native carnivores with high retention rates [94]. *Amphistegina lobifera* ranked third among species impacting shallow soft substrates. This invasive foraminifera can dominate sediment communities and introduce significant shifts in community composition [95,96] or even alter habitat structure by creating a novel habitat through the accumulation of its shells [97]. 

Seagrass meadows were assessed as the third most severely impacted habitat type. Six ICAS were identified to negatively affect this habitat type with the red algae *L. lallemandii* and *W. setacea* assessed as the most impactful. These invasive rhodophytes can form dense epiphytic mats on *Posidonia oceanica* meadows and affect their fitness [98,99,100] and the associated communities that inhabit the meadow [101,102]. The pelagic habitat was exclusively impacted by *M. leidyi*. This invasive ctenophore preys opportunistically on planktivorous organisms [103,104] and can adversely impact the structure of zooplanktonic communities [105].

Applying the CIMPAL index in a Mediterranean scale, Katsanevakis et al. [13] identified Greece as the second most heavily affected Mediterranean country by ICAS impacts, when considering the Greek waters of both the Aegean and Ionian Seas. The authors estimated CIMPAL scores by using presence/absence data for both habitat extent and ICAS population state in a Mediterranean grid of 10 × 10 km cells. In contrast, the current study employed habitat mapping data, and was conducted at a much finer scale, using a grid cell size of approximately 1 km^2^, as opposed to the 100 km^2^ cell used by Katsanevakis et al. [13]. Using just the presence of habitats instead of their actual extent in the grid cells can lead to substantial biases in CIMPAL scores, as habitats with very low coverage will equally contribute to the estimation of CIMPAL scores as the dominant habitats. Alongside the compilation of ICAS distribution data, synthesized from an exhaustive number of sources exclusively in the Aegean Sea over the past 20 years [39], the cumulative impacts estimates presented here should be considered more accurate for this specific ecoregion. 

Katsanevakis et al. [13] reported a higher CIMPAL score in coastal habitats of the Aegean, particularly in the South, which aligns with the findings of the present study. Both studies illustrated a decreasing gradient of impacts in the open sea from the North to the South; although, different species were identified as the causative agents. Katsanevakis et al. [13] attributed these impacts to the dinoflagellate *Gymnodinium catenatum* and the haptophyte *Phaeocystis pouchetii*, which have been linked to negative changes in the pelagic habitat’s food web due to the formation of toxic blooms [19]. In the present study, these two cryptogenic species were excluded due to uncertainties regarding their origin, and *M. leidyi* was the only species that impacted the pelagic habitat. 

Furthermore, both studies identified the worst invasive species in the Mediterranean and the Aegean using the same impact indicators. Both studies assessed *C. cylindracea* as the most impactful species in terms of the sum of impact scores through indicator D3. However, in Katsanevakis et al. [13], all species within the top six ranks of this indicator were macrophytes, whereas the present study included three macrophytes, two fish, and one invertebrate. In terms of indicator D4, the two studies shared three common species within the top ten rankings. 

The CIMPAL index has been effectively used as an impact assessment tool for alien species in European Seas [13,106] and inland waters [107]. One of the concerns in applying the CIMPAL index is related to the quality of habitat mapping and assessments of population state [13]. In its present application for the Aegean Sea, using a 0.01° latitude/longitude grid in combination with high-resolution shallow water habitat maps was sufficient to depict spatial patterns. The impact of each studied species was assessed based on its recorded impacts within the Mediterranean [12]. The impact weights used were based on the worst documented impacts for each species, irrespective of the location and timing of the report. However, ICAS impacts vary in space and time, and the fact that no spatiotemporal impact variance has been considered may have led to an overestimation of ICAS impacts. Finally, the current impact assessment utilizes a list of impactful ICAS derived from a recent Mediterranean review [12], which refers to documented impacts in the literature; unpublished impacts may have been missed.

This study represents the first comprehensive evaluation of ICAS’ cumulative adverse impacts on the marine habitats of the Aegean Sea. The EU Regulation 1143/2014 stipulates specific measures “to prevent, minimize and mitigate the adverse impact on biodiversity of the introduction and spread within the Union, both intentional and unintentional, of invasive alien species”. Article 19 of the regulation pertains to managing widely spread invasive alien species of Union concern, and it mandates that the management measures should be proportional to the severity of the environmental impact. Greece enforced the regulation in February 2021. It was soon realized that a national list of IAS (HELLAS-ALIENS) should be supported by a database with all important information about species origin, traits, status, habitats, pathways of introduction, potential impacts, and geographical distribution. All marine species included in HELLAS-ALIENS [108] were considered in this study. Therefore, this study provides an essential foundation for future management and mitigation actions concerning ICAS in the Aegean Sea. It identifies the most heavily impacted areas, the most affected habitats, and the most impactful ICAS in the region, through the application of the CIMPAL index and its associated indicators.

## 5. Conclusions

The application of the CIMPAL index in this study indicates that the marine ecosystems of the Aegean Sea are substantially impacted by biological invasions. These impacts were found to be more pronounced in coastal habitats, with the highest CIMPAL scores more frequently occurring in the South Aegean than the North Aegean. The ICAS’ impacts were depicted with varying magnitudes and patterns depending on the habitat examined. Shallow hard substrates (0–60 m) were assessed as the most heavily impacted habitat type, followed by shallow soft substrates (0–60 m) and seagrass meadows. The study also identified the most impactful ICAS species, both overall and within each habitat type. As an ecoregion of the eastern Mediterranean, the Aegean Sea is particularly vulnerable to biological invasions and the impacts of climate change, while also harboring invaluable biodiversity that requires conservation. This study provides a valuable tool that aligns with relevant European guidelines, aiding in the prioritization of future management actions and the implementation of mitigation measures to address biological invasions in the Aegean Sea.

## Figures and Tables

**Table 1:** List of the targeted impactful cryptogenic and alien species (ICAS) and their records in the Aegean Sea, for the application of the CIMPAL index.

Biotic Group	Phylum: Class	Species	Status	Number of Records
Macrophytes	Ochrophyta: Phaeophyceae	*Stypopodium schimperi*	Alien	67
Macrophytes	Chlorophyta: Ulvophyceae	*Caulerpa cylindracea*	Alien	283
Macrophytes	Chlorophyta: Ulvophyceae	*Codium fragile*	Alien	22
Macrophytes	Rhodophyta: Florideophyceae	*Asparagopsis* spp.	Alien	117
Macrophytes	Rhodophyta: Florideophyceae	*Lophocladia lallemandii*	Alien	224
Macrophytes	Rhodophyta: Florideophyceae	*Womersleyella setacea*	Alien	24
Macrophytes	Tracheophyta: Magnoliopsida	*Halophila stipulacea*	Alien	132
Invertebrates	Foraminifera: Globothalamea	*Amphistegina lobifera*	Alien	26
Invertebrates	Ctenophora: Tentaculata	*Mnemiopsis leidyi*	Alien	77
Invertebrates	Cnidaria: Anthozoa	*Oculina patagonica*	Cryptogenic	51
Invertebrates	Bryozoa: Gymnolaemata	*Amathia verticillata*	Cryptogenic	19
Invertebrates	Mollusca: Bivalvia	*Brachidontes pharaonis*	Alien	35
Invertebrates	Mollusca: Bivalvia	*Pinctada radiata*	Alien	159
Invertebrates	Mollusca: Gastropoda	*Conomurex persicus*	Alien	129
Invertebrates	Annelida: Polychaeta	*Hydroides elegans*	Alien	24
Invertebrates	Arthropoda: Malacostraca	*Callinectes sapidus*	Alien	115
Invertebrates	Chordata: Ascidiacea	*Styela plicata*	Alien	21
Fish	Chordata: Teleostei	*Fistularia commersonii*	Alien	171
Fish	Chordata: Teleostei	*Lagocephalus sceleratus*	Alien	254
Fish	Chordata: Teleostei	*Parupeneus forsskali*	Alien	70
Fish	Chordata: Teleostei	*Pempheris rhomboidea*	Alien	31
Fish	Chordata: Teleostei	*Pterois miles*	Alien	574
Fish	Chordata: Teleostei	*Sargocentron rubrum*	Alien	88
Fish	Chordata: Teleostei	*Siganus luridus*	Alien	919
Fish	Chordata: Teleostei	*Siganus rivulatus*	Alien	748
Fish	Chordata: Teleostei	*Upeneus pori*	Alien	23

## Data Availability

The data presented in this study are available on request from the corresponding author.

## Supplementary Materials

The following supporting information can be downloaded at: https://www.mdpi.com/article/10.3390/biology12070933/s1, Table S1: Matrix of impact weights wi,j for all combinations of the targeted 26 impactful cryptogenic and alien species and the 10 habitat types. Figures S1–S10: Distribution of the ten habitat types in the Aegean Sea. Figure S11: Impact weight table. Figure S12: Strength of evidence types and impact magnitude categories. References [109,110,111,112,113,114,115,116,117,118,119,120,121,122,123,124,125,126,127,128,129,130,131,132,133,134,135,136,137,138,139,140,141,142,143,144,145,146,147,148,149,150,151,152,153,154,155,156,157] are cited in the Supplementary Materials.
