# Supplementary material for: Cumulative Negative Impacts of Invasive Alien Species on Marine Ecosystems of the Aegean Sea
Source: Biology (Basel). 2023 Jun 29;12(7):933. doi: 10.3390/biology12070933 (PMC10376206; doi:10.3390/biology12070933)
Supplement: Supplementary file 1 [file biology-12-00933-s001.zip › biology-2400427-supplementary/Figure S1-S12.pdf]

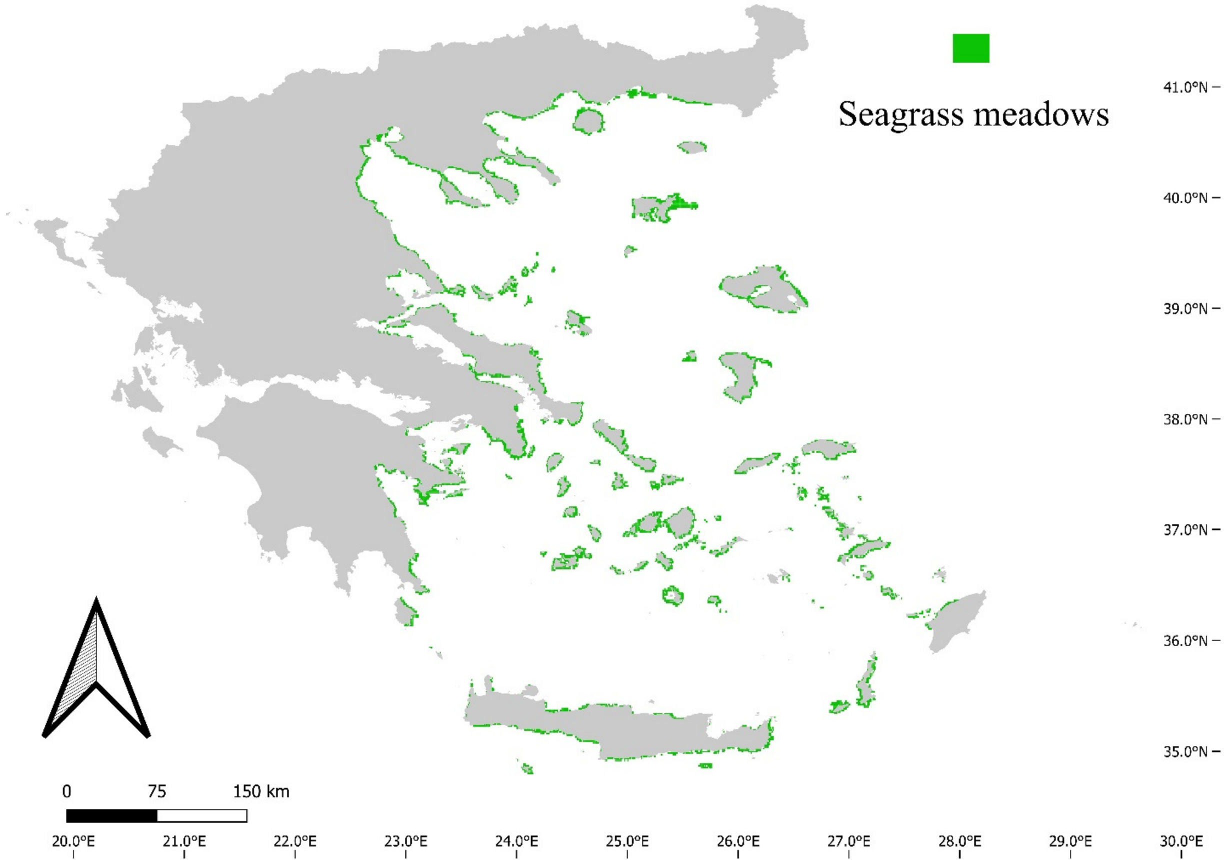

Figure S1 Distribution of Seagrass meadows in the Aegean Sea.

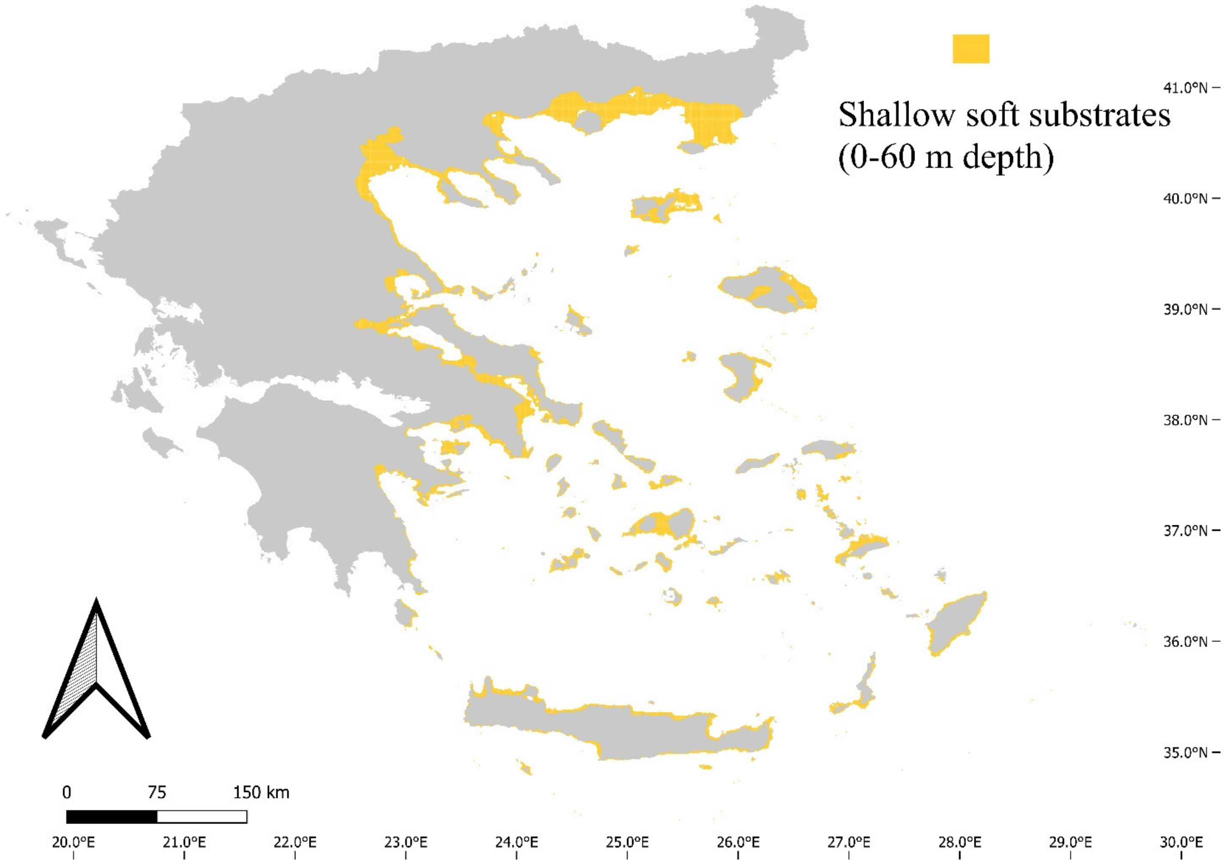

Figure S2 Distribution of shallow soft substrates (0–60 m depth) in the Aegean Sea.

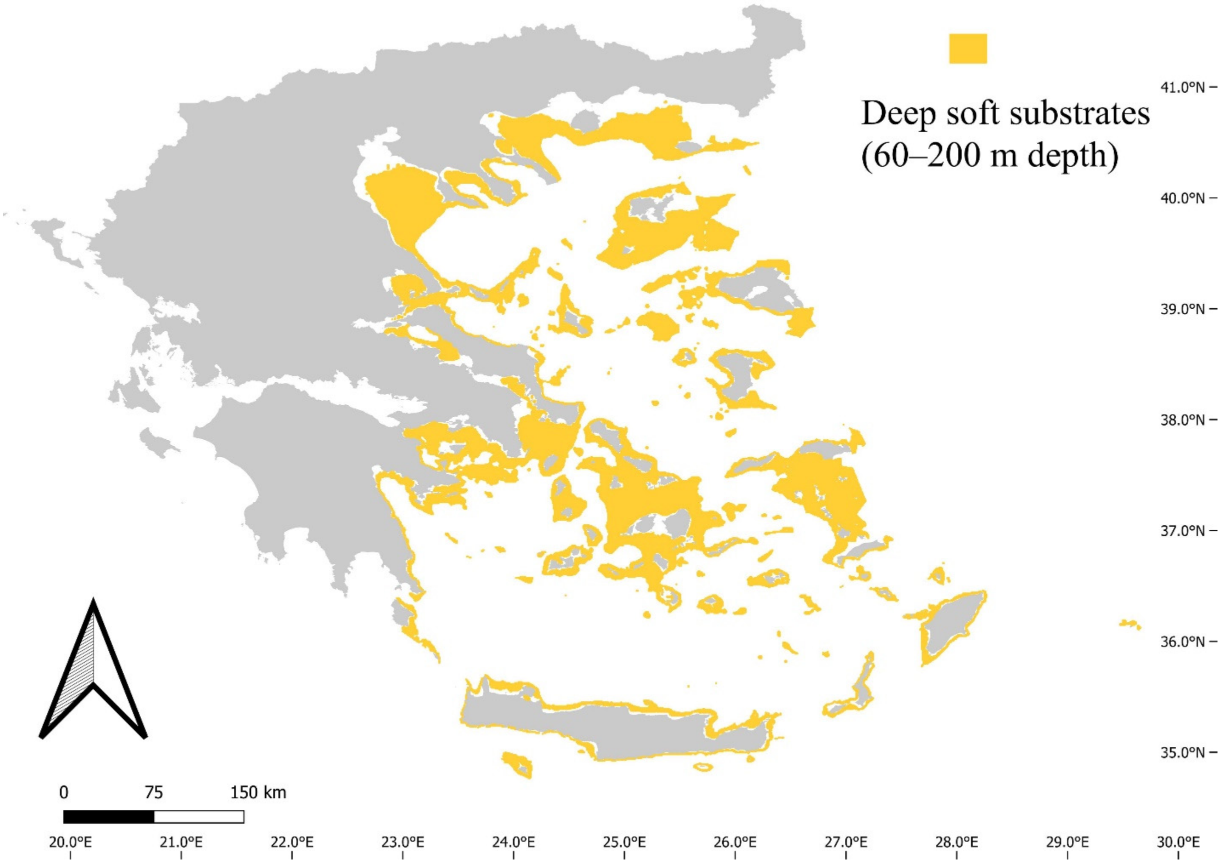

Figure S3 Distribution of deep soft substrates (60–200 m depth) in the Aegean Sea.

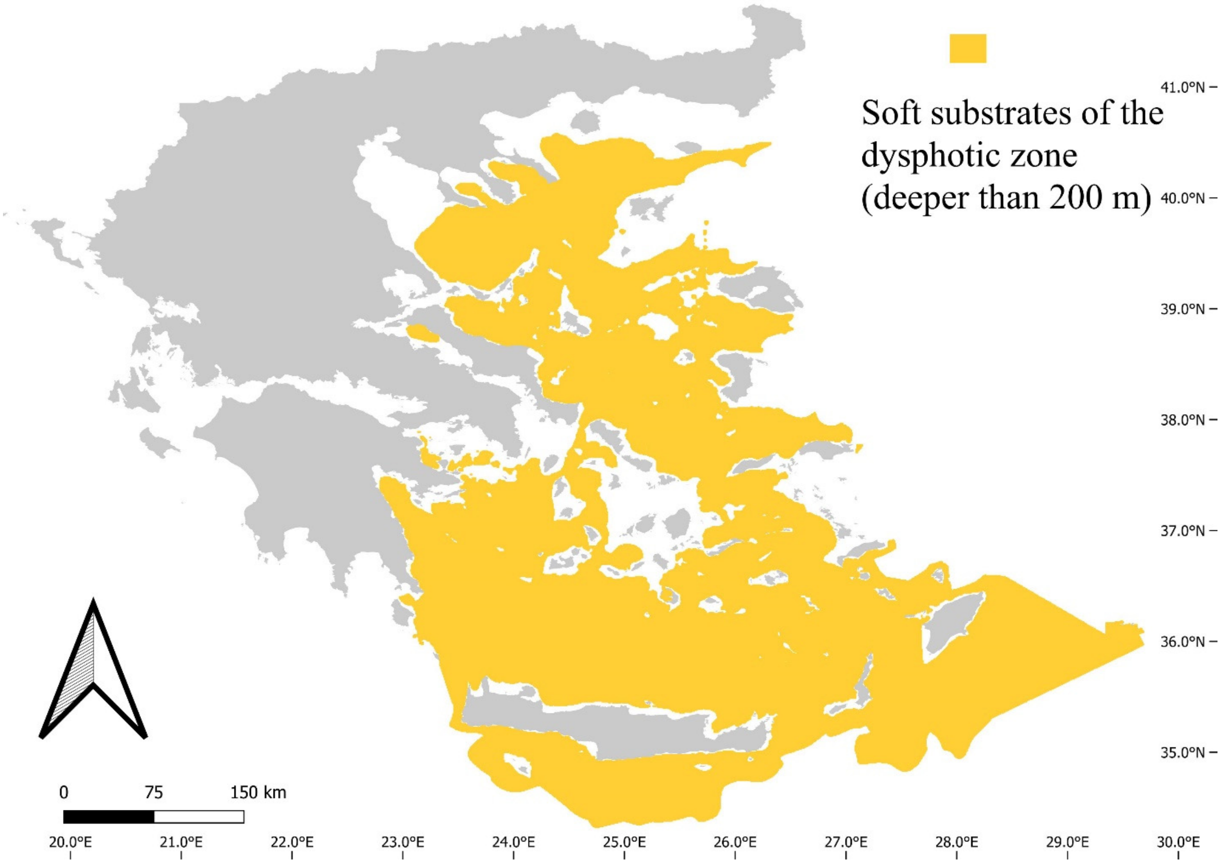

Figure S4 Distribution of soft substrates of the dysphotic zone (deeper than 200 m depth) in the Aegean Sea.

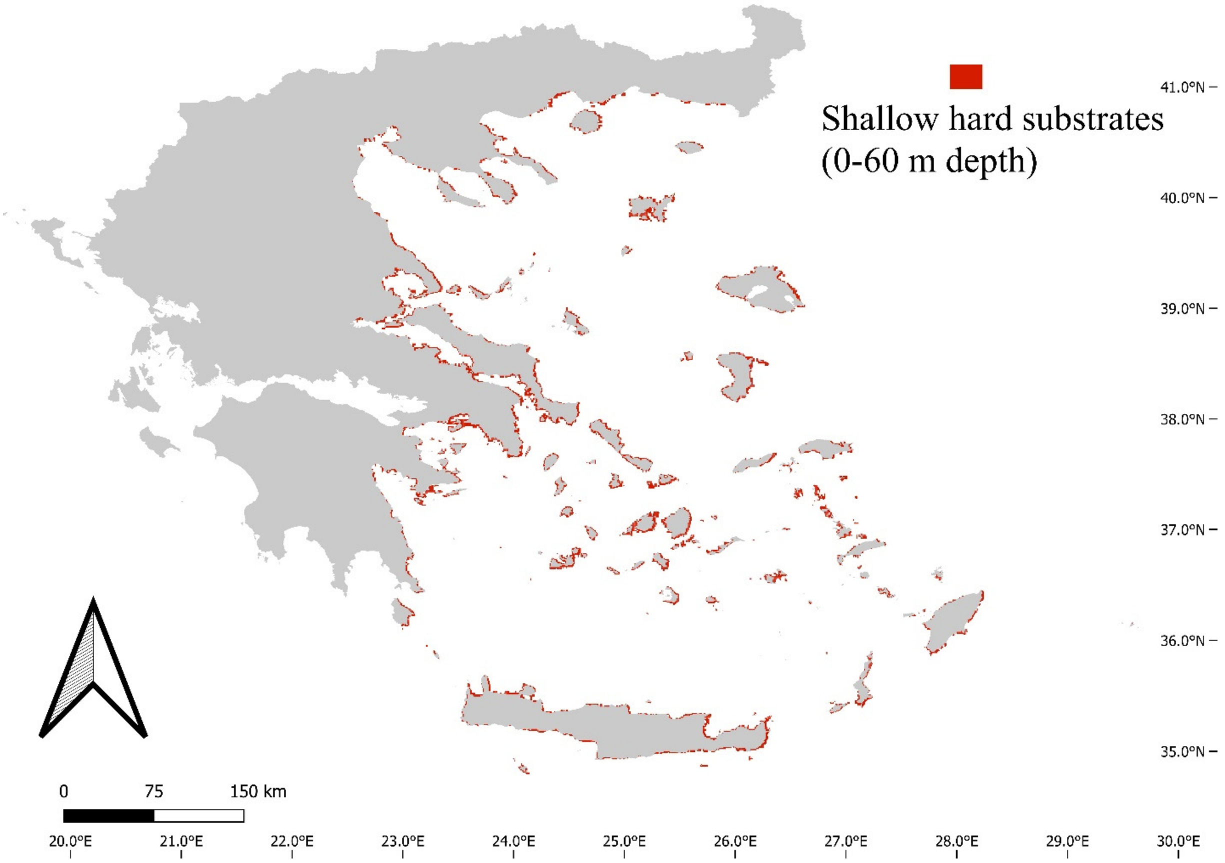

Figure S5 Distribution of shallow hard substrates (0-60 m depth) in the Aegean Sea.

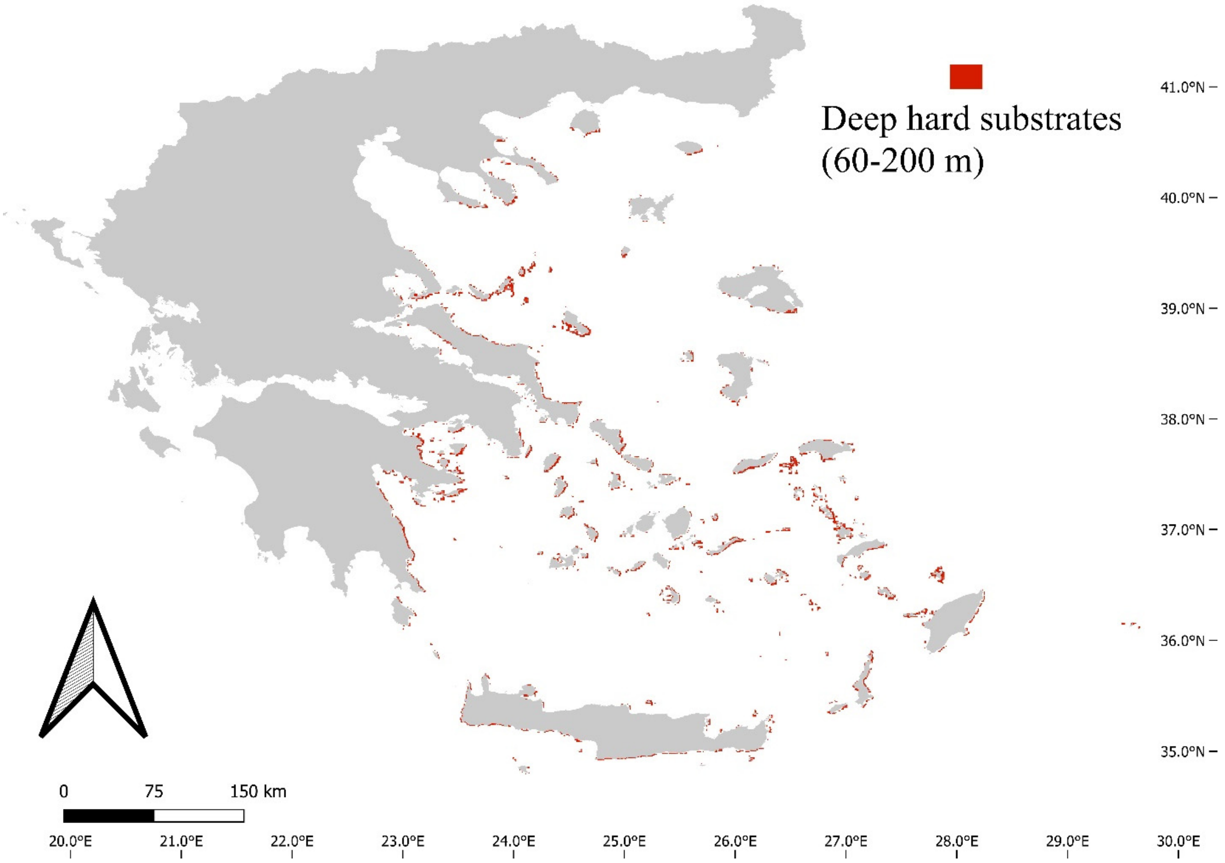

Figure S6 Distribution of deep hard substrates (60-200 m) in the Aegean Sea.

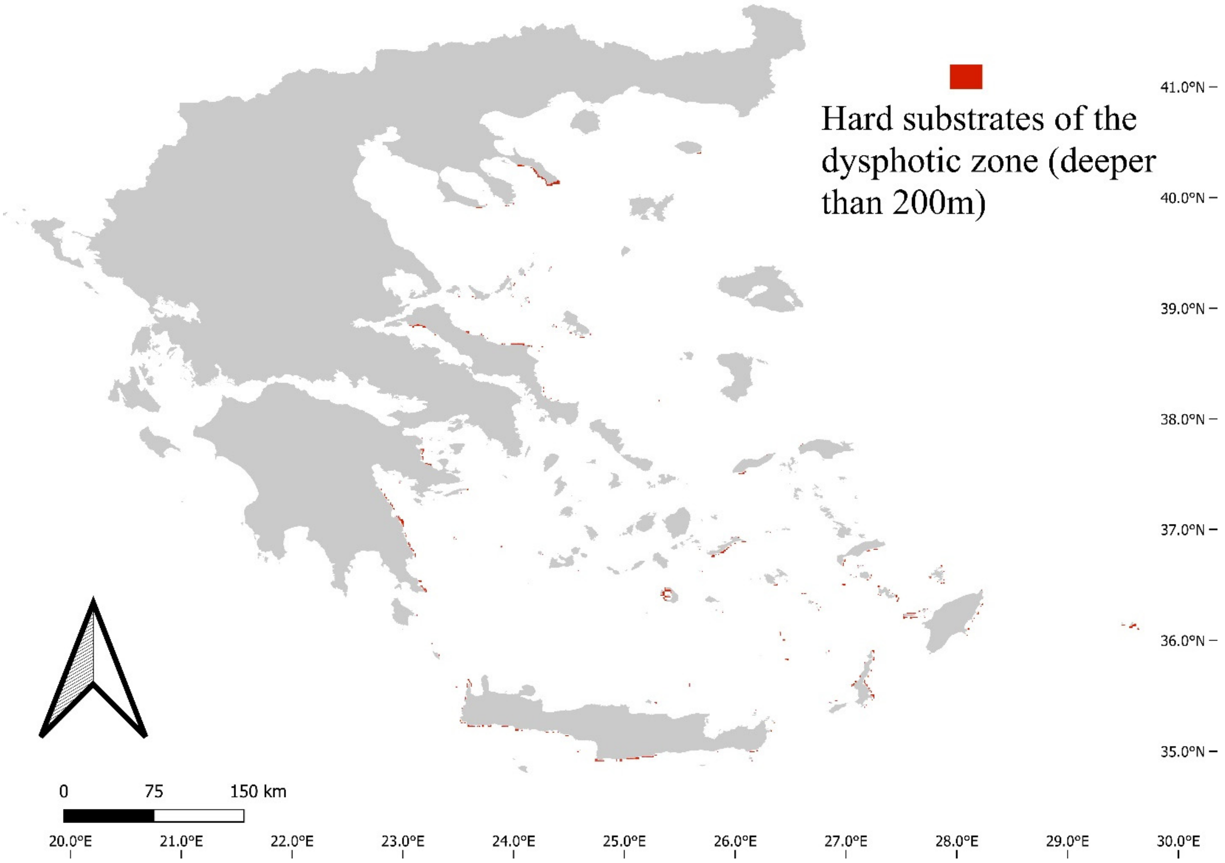

Figure S7 Distribution of hard substrates of the dysphotic zone (deeper than 200 m) in the Aegean Sea.

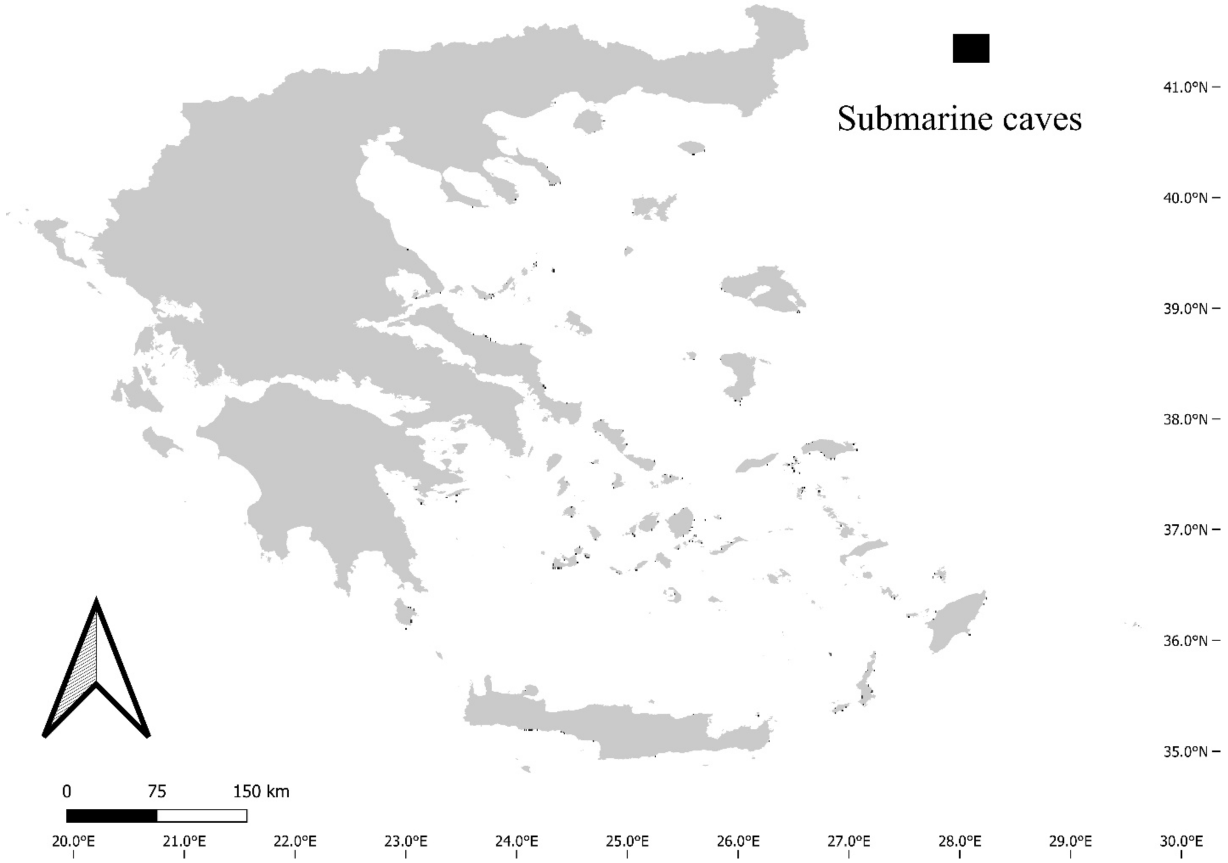

Figure S8 Distribution of submarine caves in the Aegean Sea.

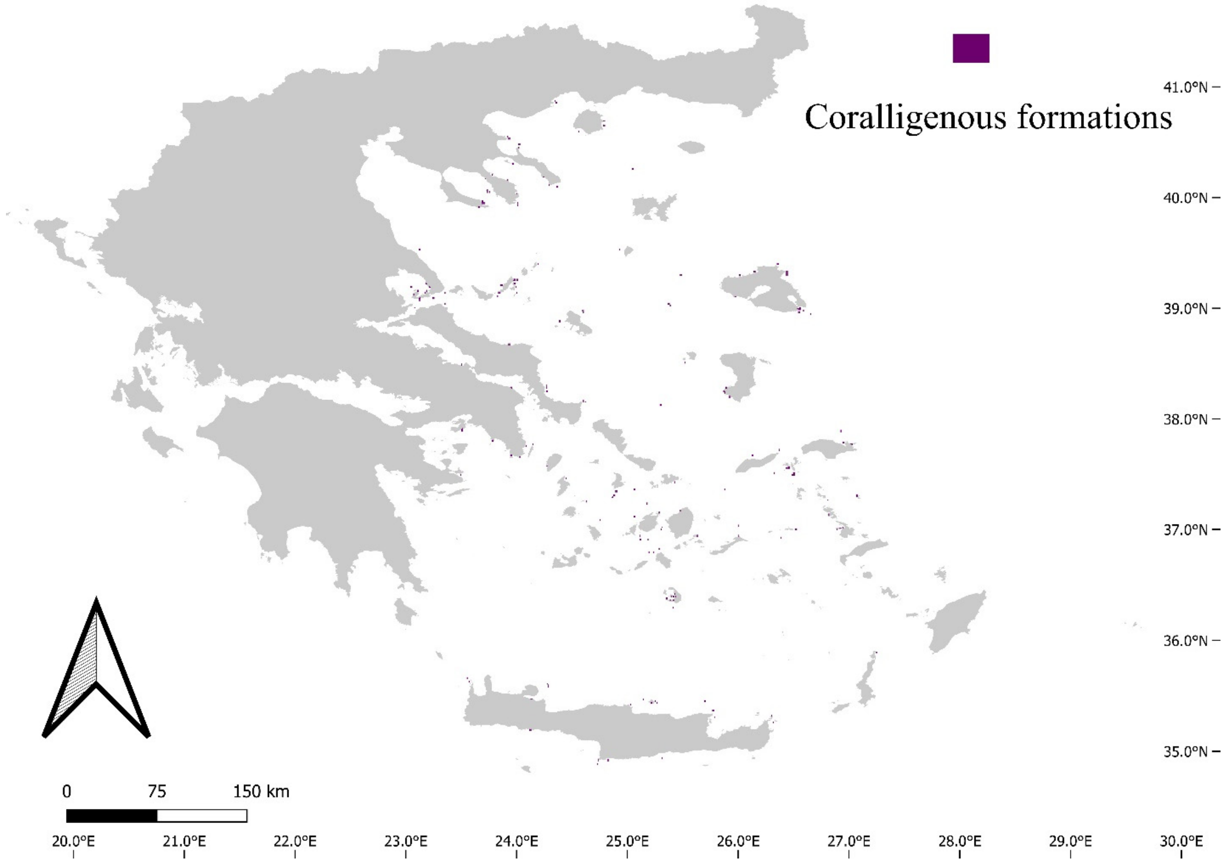

Figure S9 Distribution of coralligenous formations in the Aegean Sea.

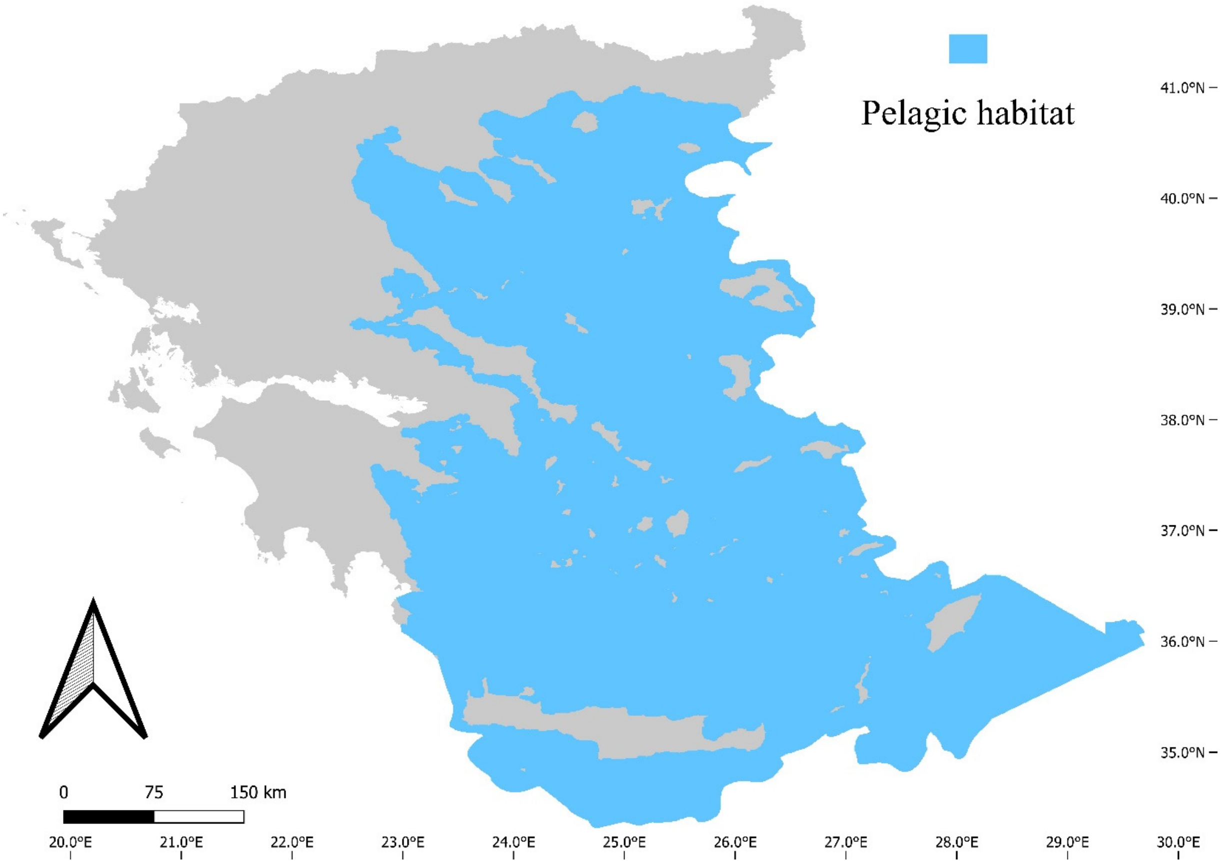

Figure S10 Distribution of the pelagic habitat in the Aegean Sea.

		Impact magnitude				
		Minimal	Minor	Moderate	Major	Massive
Strength of evidence	Robust	0	1	2	4	8
	Medium	0	0	1	2	4
	Limited	0	0	0	1	2

Figure S11 Impact weights $w_{i,j}$ for species i at habitat j based on impact magnitude and strength of evidence, as defined in Figure S12 (based on [13,19]).

Strength of evidence	
**Robust**	The impact is estimated by experimental studies. The different types of experiments are **Manipulative Experiments** (field and lab experiments based on a random selection of experimental units and include treatments and controls) and **Natural Experiments** (field experiments based on natural selection of experimental units).
**Medium**	Impact assessment is derived from **Direct Observations** (direct observations or measurements of impact without doubt), **Modelling** (ecosystem models that simulate alien species impacts on biodiversity) and **non-experimental based correlations** (significant correlations between an alien species and an impact on native biodiversity, not based on an experimental design).
**Limited**	Qualitative Impact assessment based on **Expert Judgement** through the empirical knowledge of experts based on alien species' traits.
**Impact magnitude**	
**Massive**	Changes in community composition through local or global extinction (negative) because of competition, predation, grazing, parasitism, toxicity or bio-fouling; impact on ecosystem processes and ecosystem functioning resulting in species composition changes; or ecological engineering, resulting in change in community composition. Massive magnitude applies when under the hypothetical scenario of the alien species extinction, the induced changes are considered as irreversible within 10 years or within 3 generations of the extinct native taxon/a, whichever is longer.
**Major**	Changes in community composition through local or global extinction (negative) because of competition, predation, grazing, parasitism, toxicity or bio-fouling; impact on ecosystem processes and ecosystem functioning resulting in species composition changes; or ecological engineering, resulting in change in community composition. Major magnitude applies when under the hypothetical scenario of the alien species extinction, the induced changes are considered as reversible within 10 years or within 3 generations of the extinct native taxon/a, whichever is longer.
**Moderate**	Native species population changes due to competition, predation, grazing, parasitism, toxicity or bio-fouling, but without changes in community composition; or impact on ecosystem processes and ecosystem functioning resulting in population declines but no substantial change in community composition; or ecological engineering, resulting in population declines but no substantial change in community composition.
**Minor**	Impact on individual fitness of a native species due to competition, predation, grazing, parasitism, toxicity or bio-fouling without substantial population changes; minor impact on ecosystem processes and ecosystem functioning with no related population changes; any environmental or habitat alterations in chemical, physical or structural characteristics do not result in population changes.
**Minimal**	No effect on fitness of native species; negligible impact on native species due to competition, predation, grazing, parasitism, toxicity or bio-fouling; no effects on ecosystem processes and ecosystem functioning; no chemical, physical or structural impact on the ecosystem (not an ecosystem engineer).

Figure S12 Strength of evidence types (based on Katsanevakis et al., 2014) and impact magnitude categories (based on Blackburn et al., 2014; Volery et al., 2020).
